# Correlations of Myeloperoxidase (MPO), Adenosine deaminase (ADA), C–C motif chemokine 22 (CCL22), Tumour necrosis factor alpha (TNFα) and Interleukin-6 (IL-6) mRNA expression in the nasopharyngeal specimens with the diagnosis and severity of SARS-CoV-2 infections

**DOI:** 10.1080/22221751.2022.2157338

**Published:** 2023-01-02

**Authors:** Kelvin Hei-Yeung Chiu, Cyril Chik-Yan Yip, Rosana Wing-Shan Poon, Kit-Hang Leung, Xin Li, Ivan Fan-Ngai Hung, Kelvin Kai-Wang To, Vincent Chi-Chung Cheng, Kwok-Yung Yuen

**Affiliations:** a Department of Microbiology, Queen Mary Hospital, Pokfulam, Hong Kong Special Administrative Region, People’s Republic of China; b State Key Laboratory for Emerging Infectious Disease, Carol Yu Centre for Infection, Department of Microbiology, Li Ka Shing Faculty of Medicine, The University of Hong Kong, Hong Kong Special Administrative Region, People’s Republic of China; c Department of Microbiology, School of Clinical Medicine, Li Ka Shing Faculty of Medicine, The University of Hong Kong, Pokfulam, Hong Kong Special Administrative Region, People's Republic of China; d Department of Medicine, School of Clinical Medicine, Li Ka Shing Faculty of Medicine, The University of Hong Kong, Pokfulam, Hong Kong Special Administrative Region, People’s Republic of China; e Department of Infectious Disease and Microbiology, The University of Hong Kong-Shenzhen Hospital, Shenzhen, People’s Republic of China; f Centre for Virology, Vaccinology and Therapeutics, , Hong Kong Science and Technology Park, Pak Shek Kok, Hong Kong Special Administrative Region, China

**Keywords:** Nasopharyngeal specimen, COVID-19 severity, myeloperoxidase, adenosine deaminase, CCL22

## Abstract

Cytokine dynamics in patients with coronavirus disease 2019 (COVID-19) have been studied in blood but seldomly in respiratory specimens. We studied different cell markers and cytokines in fresh nasopharyngeal swab specimens for the diagnosis and for stratifying the severity of COVID-19. This was a retrospective case-control study comparing Myeloperoxidase (MPO), Adenosine deaminase (ADA), C–C motif chemokine ligand 22 (CCL22), Tumour necrosis factor alpha (TNFα) and Interleukin-6 (IL-6) mRNA expression in 490 (327 patients and 163 control) nasopharyngeal specimens from 317 (154 COVID-19 and 163 control) hospitalized patients. Of the 154 COVID-19 cases, 46 died. Both total and normalized MPO, ADA, CCL22, TNFα, and IL-6 mRNA expression levels were significantly higher in the nasopharyngeal specimens of infected patients when compared with controls, with ADA showing better performance (OR 5.703, 95% CI 3.424–9.500, *p *< 0.001). Receiver operating characteristics (ROC) curve showed that the cut-off value of normalized ADA mRNA level at 2.37 × 10^–3^ had a sensitivity of 81.8% and specificity of 83.4%. While patients with severe COVID-19 had more respiratory symptoms, and elevated lactate dehydrogenase, multivariate analysis showed that severe COVID-19 patients had lower CCL22 mRNA (OR 0.211, 95% CI 0.060–0.746, *p* = 0.016) in nasopharyngeal specimens, while lymphocyte count, C-reactive protein, and viral load in nasopharyngeal specimens did not correlate with disease severity. In summary, ADA appears to be a better biomarker to differentiate between infected and uninfected patients, while CCL22 has the potential in stratifying the severity of COVID-19.

## Introduction

The pandemic of coronavirus disease 2019 (COVID-19) caused by severe acute respiratory syndrome coronavirus 2 (SARS-CoV-2) has affected almost 600 million people with over 6 million deaths as of 28 August 2022 [1]. COVID-19 is primarily a respiratory disease with a wide range of pulmonary and extrapulmonary manifestations which ranges from asymptomatic virus shedding to respiratory and multiorgan failure with bilateral multifocal ground glass opacities with patchy consolidations in the lung [2]. Various manifestations associated with severe COVID-19 are believed to be related to the immune-dysregulation and hyper-inflammatory state of the patient [3–6]. Therefore, the temporal profile of inflammatory cytokines and chemokines of COVID-19 becomes the centre of attention.

Studies on dynamics of cytokines and inflammatory markers in patients with COVID-19 of different severity were reported in blood [7–10], but seldomly in nasopharyngeal specimens [11]. As the use of nasopharyngeal specimens remains the gold standard for diagnosis of COVID-19 [12,13], these specimens are readily available in the laboratory for further investigations. They provide an opportunity to investigate the differences in local inflammatory response mounted by various cell types in the nasopharyngeal mucosa of COVID-19 patients.

Pro-inflammatory cytokines, tumour necrosis factor alpha (TNFα), and interleukin-6 (IL-6), are widely studied in SARS-CoV-2 infection. Studies have shown that TNFα and IL-6 are highly elevated in the blood of patients with severe COVID-19 [14]. IL-6 has also been implicated as the cause of cytokine release syndrome in SARS-CoV-2 infection, and hence the use of IL-6 inhibitors tocilizumab for the management of severe COVID-19 [15]. Three additional markers were chosen as the targets of our one-step real-time RT-qPCR (reverse transcription-quantitative polymerase chain reaction), including myeloperoxidase (MPO), adenosine deaminase (ADA), and C–C motif chemokine 22 (CCL22). MPO is present in polymorphonuclear cells and is highly expressed during early innate immune response, as it produces hypochlorous acid for intracellular killing. ADA is a product of activated lymphocytes. Mutation of the ADA gene has been linked to primary immunodeficiency such as severe combined immunodeficiency, which leads to depletion of lymphocytes and hence impairment of both cellular and humoral immunity [16]. CCL22, also known as macrophage-derived chemokine, is secreted by several types of immune cells, mainly activated macrophages and dendritic cells [17–19]. It is induced by lipopolysaccharide (LPS), IL-4, and IL-13, and in T cells by T cell receptor (TCR) stimulation [20], but downregulated by Th1-type cytokines, such as IFNγ [21]. CCL22 causes chemotactic migration of dendritic cells and Th2 cells, thus plays an important role in the recruitment of Th2 cells into the inflammatory sites and the regulation of Th2-related immune responses.

With the upsurge in number of COVID-19 patients during the fifth wave mainly due to Omicron variant in Hong Kong [22–24], we here reported the use of one-step real-time RT-qPCR on nasopharyngeal specimens to investigate the role of nasopharyngeal expression of MPO, ADA, CCL22, TNFα, and IL-6 messenger RNA (mRNA) in the diagnosis of COVID-19. We also compared the differences in the expression profile in patients with mild and severe COVID-19 in relationship to the clinical and laboratory findings of hospitalized patients.

## Materials and methods

### Study design and patient selection

This was a multi-centre, retrospective cohort study in a healthcare region in the Hong Kong West, including an university-affiliated teaching hospital (Queen Mary Hospital of 1700-bed) and another three extended care hospitals (bed number ranges from 270 to 530). Adult patients (age ≥ 18) admitted to our healthcare region from 26th February to 1st November, 2022 with laboratory-confirmed COVID-19 and at least one archived RNA extracts from nasopharyngeal specimens were included in this study. A confirmed case of COVID-19 was defined by the detection of SARS-CoV-2 RNA by LightMix E-gene RT–PCR kit (TIB Molbiol, Berlin, Germany) [25]. First diagnosis was defined by the date of detection of SARS-CoV-2 RNA by RT–PCR or SARS-CoV-2 antigen by rapid antigen test using lateral flow immunochromatographic assays (GLINE-2019-nCoV Ag, YHLO, China). Adult patients with a negative SARS-CoV-2 RT–PCR on their nasopharyngeal swabs with no recent COVID-19 diagnosed within 1 month were included as control. This study was approved by the Institutional Review Board of the University of Hong Kong/Hospital Authority Hong Kong West Cluster (UW 22-052).

### Covariates of interest

Demographic data, medical comorbidities, presenting symptoms, oxygen saturation, haematological and biochemical parameters on admission, together with vaccination status were retrieved from the electronic health records. The two COVID-19 vaccines available in Hong Kong are BNT162b2 (Pfizer and BioNTech) and CoronaVac whole-virion inactivated vaccine (Sinovac Life Sciences). Only vaccination received at least 14 days prior to infection is considered as valid in this study. Patients were considered fully vaccinated when they received the required number of vaccines in the primary series (i.e. 2 doses of BNT162b2 or 3 doses of CoronaVac). Viral load as expressed by the PCR cycle threshold (Ct) value was also obtained from our laboratory information system. Cardiovascular disease was defined as the presence or history of cardiomyopathy, congestive heart failure, or coronary artery disease. Chronic lung disease was defined as chronic obstructive pulmonary disease, bronchiectasis, or asthma. Immunosuppressant was defined as corticosteroid, steroid-sparing agents, and chemotherapy. We defined severe disease as oxygen saturation (SpO_2_) < 94% on room air on admission, admission to the intensive care unit (ICU) due to COVID-19, or death as from previous study [26].

### One step real-time RT-qPCR on MPO, ADA, CCL22, TNFα, and IL-6 mRNA detection

MPO, ADA, CCL22, TNFα, and IL-6 mRNA levels were measured by one-step real-time RT-qPCR with or without normalization with Glyceraldehyde 3-phosphate dehydrogenase (GAPDH) RNA levels in the nasopharyngeal swabs (primer sequences in Supplementary Table 1) for comparison between COVID-19 cases and controls, or between mild and severe COVID-19 cases.

Specimens were freshly extracted on arrival at the laboratory because storing specimens at refrigerator temperature without lysis buffer or RNA extraction would lead to mRNA marker loss in our pilot study using 104 and 62 specimens extracted immediately and more than 1 d after collection respectively (Supplementary Table 2). RNA was extracted using MagNA Pure 96 extraction system (Roche, Basel, Switzerland), 200 μl of each specimen was subjected to nucleic acid extraction and 50 μl of eluate was obtained. In-house one-step real-time RT-qPCR assays targeting MPO, ADA, CCL22, TNFα, IL-6, and GAPDH were performed. A reagent mixture (20 μl) contained 10 μl of 2x QuantiNova Probe RT–PCR Master Mix, 0.2 μl of QN Probe RT-Mix, 1.6 μl of each 10 μM forward and reverse primer, 0.4 μl of 10 μM probe, 1.2 μl of RNase-free water and 5 μl of RNA as the template. The thermal cycling condition was 10 min at 45°C for reverse transcription and 5 min at 95°C for PCR initial activation, followed by 45 cycles of 5 s at 95°C and 30 s at 55°C. All reactions were performed using the LightCycler 96 Real-Time PCR System (Roche). Plasmids containing target sequences were used as standards for quantification. Specimens with negative GAPDH RT–PCR were rejected as it represents poorly taken nasopharyngeal specimens. If the volume of specimen was insufficient for performing all 6 PCR reactions, priority was given to GAPDH, MPO, ADA, and CCL22.

### Statistical analysis

Descriptive statistics were reported as mean with standard deviation (SD) or median with interquartile range (IQR) depending on the distribution of continuous variables. Frequency with percentages was reported for categorical variables. Student’s *t-*test or Mann–Whitney *U* test was used for two-group comparison of continuous variables. Fisher’s exact test or Chi square test was performed for two-group comparison for categorical variables. Wilcoxon signed rank test was used for comparison between two groups of matched samples. Receiver operating characteristics (ROC) curves for each markers were plotted, and the best cut-off value, sensitivity, and specificity of each markers were determined respectively. Variables that were considered as statistically significant in univariate analysis were subjected to multivariable analysis by binomial logistic regression using backward stepwise regression to model the relationship between severity of COVID-19 and other covariates. Variables that were not available in more than 50% of any particular group of patients were excluded in multivariable analysis. Statistical analyses were performed using SPSS version 24.0 or PRISM version 6.0. A *p*-value less than 0.05 was considered as statistically significant.

## Results

**Figure 1.**
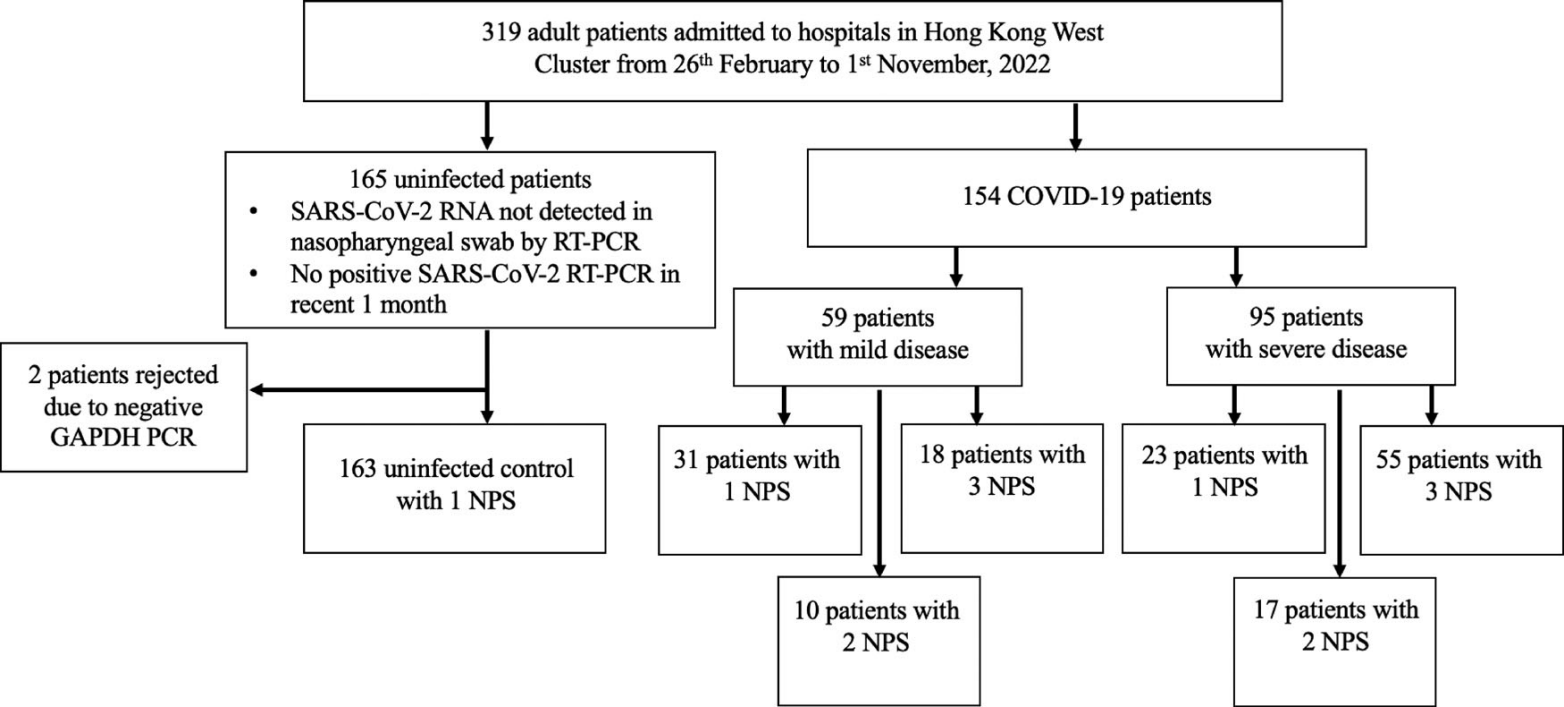
Flowchart of patient inclusion in this study. Severe disease is defined as the need for supplemental oxygen on admission, admission to the intensive care unit (ICU) due to COVID-19, or death. Abbreviations: NPS, Nasopharyngeal specimens.

Between 26th February to 1st November 2022, 154 COVID-19 and 165 uninfected patients were included in this study respectively, with 2 uninfected patients subsequently rejected due to a negative GAPDH one-step real-time RT-qPCR. Patients in the control group were admitted to the hospital due to various reasons as shown in Table 1. The flowchart of patient inclusion in this study was shown in Figure 1. Table 1 summarized the differences between the COVID-19 and control patients included in this study. The median age of all COVID-19 patients included was 82 years (IQR 69–89), which was similar to that of the control group (median 85, with IQR 71–90). The female ratios were similar between the two groups, with 50.0% in the infected group, and 58.3% in the control group. Higher prevalence of chronic liver and renal impairment, autoimmune disease and immunosuppressant use were observed in the infected group, but the prevalence of other medical comorbidities was similar. Higher mortality rate was observed in our infected patients (infected 29.9% vs uninfected 4.3%, *p* < 0.001). Blood test on admission revealed significantly higher haemoglobin level, lower lymphocyte and platelet count in our COVID-19 patients. Biochemical tests revealed lower sodium, higher potassium, urea, creatinine and alanine transaminase level in infected patients. Lactate dehydrogenase and C-reactive protein were both significantly higher in infected patients when compared with uninfected control. As only 25 (15.5%) and 80 (49.7%) of the uninfected patients received blood test for lactate dehydrogenase and C-reactive protein respectively, these parameters were not included in subsequent multivariate analysis. Vaccination status was significantly different between the two groups (*p* < 0.001).

**Table 1.** Clinical characteristics and outcome of all patients included in the study * *p* < 0.05 ** < 50% of the uninfected patients had blood taken for lactate dehydrogenase and C-reactive protein. Data are expressed as *n* (%) or median (inter-quartile range), unless otherwise stated.

	Uninfected patients	Infected patients	*p*-value
*Total number of cases*	163	154	
*Age – median (Interquartile range)*	85 (71–90)	82 (69–89)	0.191
*Female*	95 (58.3%)	77 (50.0%)	0.086
*Number of deaths*	7 (4.3%)	46 (29.9%)	<0.001*
*Past Medical History*			
* Hypertension*	95 (58.3%)	84 (54.5%)	0.289
* Diabetes Mellitus*	54 (33.1%)	53 (34.4%)	0.451
* Hyperlipidaemia*	51 (31.3%)	43 (27.9%)	0.297
* Cardiovascular disease*	31 (19.0%)	39 (25.3%)	0.112
* Chronic Renal Impairment*	27 (16.6%)	41 (26.6%)	0.020*
* Cerebral Vascular Accident*	42 (25.8%)	35 (22.7%)	0.309
* Obesity*	2 (1.2%)	1 (0.6%)	0.521
* Chronic Liver Disease*	3 (1.8%)	13 (8.4%)	0.007*
* Chronic Lung Disease*	22 (13.5%)	21 (13.6%)	0.550
* Solid Organ Malignancy*	29 (17.8%)	18 (11.7%)	0.085
* Haematological Malignancy*	7 (4.3%)	10 (6.5%)	0.268
* Solid Organ Transplantation*	3 (1.8%)	9 (5.8%)	0.057
* Haematological Transplantation*	1 (0.6%)	0 (0.0%)	0.514
* Autoimmune Disease*	1 (0.6%)	12 (7.8%)	0.001*
* Immunosuppressant*	18 (11.0%)	30 (19.5%)	0.026*
*Clinical Presentation of infected patients*			
* Fever*	–	72 (46.8%)	–
* Cough*	–	68 (44.2%)	–
* Sputum*	–	40 (26.0%)	–
* Shortness of breath*	–	83 (53.9%)	–
* Chest pain*	–	9 (5.8%)	–
* Gastrointestinal Symptoms*	–	12 (7.8%)	–
*Reasons for admission of uninfected patients*			
* Infection*	51 (31.3%)	–	–
* Cardiovascular/ Pulmonary*	25 (15.3%)	–	–
* Gastrointestinal*	8 (4.9%)	–	–
* Malignancy*	17 (10.4%)	–	–
* Metabolic*	6 (3.7%)	–	–
* Neurological*	14 (8.6%)	–	–
* Trauma*	23 (14.1%)	–	–
* Clinical admission*	19 (11.7%)	–	–
*Blood Parameters on admission – median (interquartile range)*			
* Haemoglobin, g/dL*	10.4 (8.7–12.0)	11.4 (9.4–13.1)	0.005*
* White Blood Cell,* × 10^9^/L	7.9 (6.1–10.5)	7.9 (5.1–11.9)	0.709
* Neutrophil,* × 10^9^/L	5.6 (4.0–7.9)	6.3 (3.6–9.5)	0.249
* Lymphocyte,* × 10^9^/L	1.1 (0.8–1.6)	0.8 (0.4–1.2)	<0.001*
* Platelet,* × 10^9^/L	241 (179–313)	192 (137–279)	0.001*
* Sodium, mmol/L*	138 (134–140)	135 (132–140)	0.005*
* Potassium, mmol/L*	3.8 (3.6–4.3)	4.0 (3.6–4.4)	0.020*
* Urea, mmol/L*	6.2 (4.8–10.0)	8.8 (5.5–17.6)	<0.001*
* Creatinine, mmol/L*	82 (59–117)	107 (72–179)	<0.001*
* Alkaline Phosphatase, U/L*	83 (66–107)	83 (67–123)	0.494
* Alanine Transaminase, U/L*	16 (11–27)	25 (16–41)	<0.001*
Lactate Dehydrogenase**, *U/L*	252 (202–293)	348 (256–497)	<0.001*
C-reactive protein**, *mg/dL*	1.9 (0.8–7.8)	6.5 (1.8–11.9)	0.001*
*Vaccination Status*			<0.001*
* Complete*	93 (57.1%)	42 (27.3%)	
* Incomplete/Unvaccinated*	70 (42.9%)	112 (72.7%)	

In total, 490 (327 patients and 163 control) nasopharyngeal specimens were taken from 317 (154 COVID-19 and 163 control) hospitalized patients, with 1 nasopharyngeal swab from each control patient, and a mean of 2.12 nasopharyngeal specimens per COVID-19 patient. Specimens were taken at different time points after admission, with the median (IQR) time of specimen collection being 10 days (4.0–17.5 days) post diagnosis. The median (IQR) time difference between the first and second and between second and third specimen collections were both 2.0 days (1.0–2.0 days). The distribution of specimen collection time calculated based on the day of first diagnosis was summarized in Supplementary Figure 1. GAPDH, MPO, ADA, and CCL22 one-step real-time RT-qPCR were performed in all specimens. However, due to the limited volume of archived specimens, TNFα and IL-6 one-step real-time RT-qPCR were performed on 454 and 452 archived extracts, respectively.

**Figure 2.**
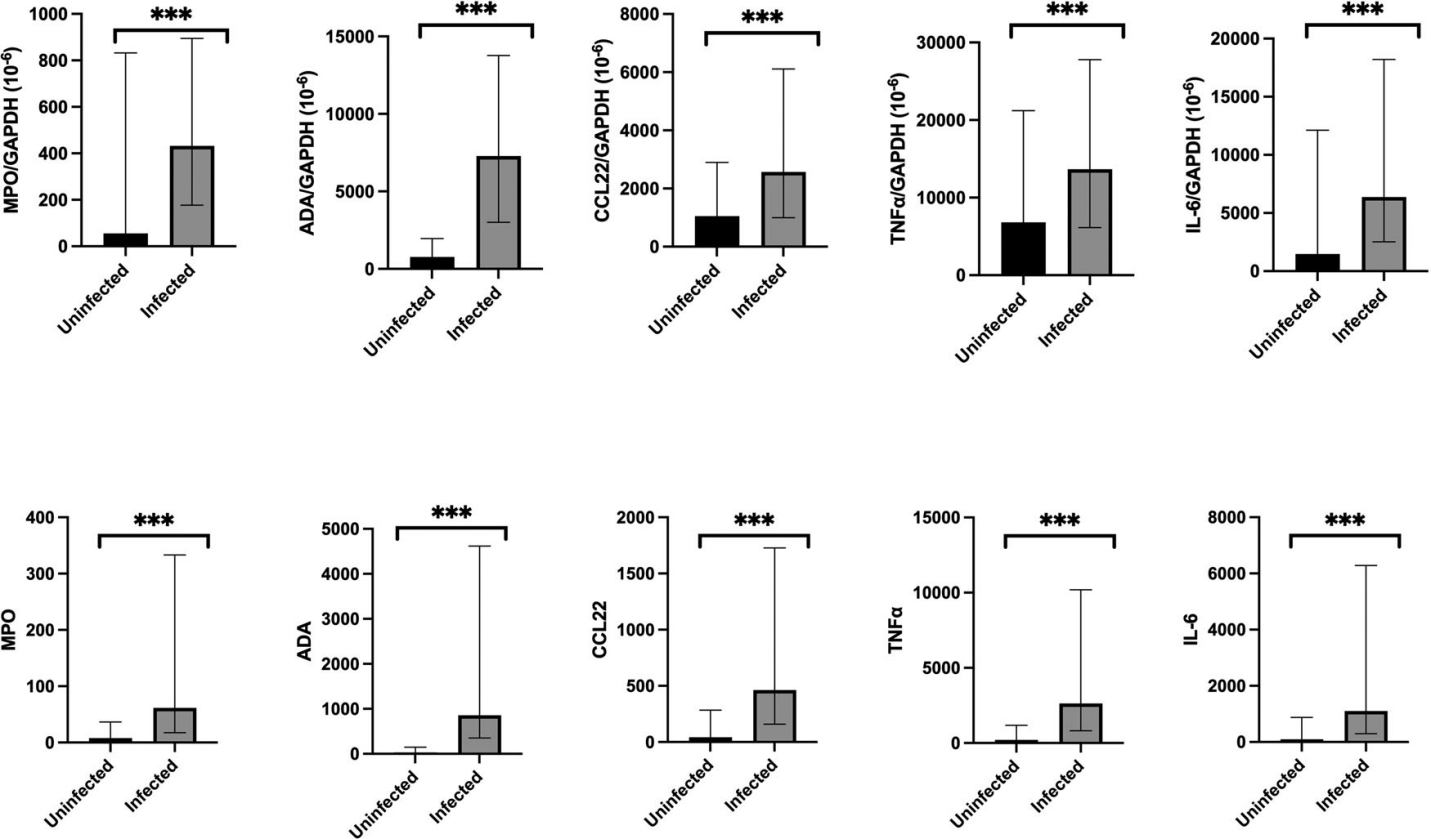
Comparison of normalized and total MPO, ADA, CCL22, TNFα, and IL-6 mRNA expression in nasopharyngeal specimens between COVID-19 infected and uninfected individuals. The bar represents the median of the samples, the hinges mark the interquartile range. *p < 0.05, **p < 0.005, ***p < 0.001.

Normalized MPO mRNA level (IQR) (infected 431.8 × 10^−6^ (177.3–894.3) vs control 55.4 × 10^−6^ (0.0–832.3), *p* < 0.001), ADA mRNA level (IQR) (infected 7283.4 × 10^−6^ (3001.8–13,768.7) vs control 763.3 × 10^−6^ (0.0–1960.2), *p* < 0.001), CCL22 mRNA level (IQR) (infected 2573.1 × 10^−6^ (994.6–6112.2) vs control 1046.9 × 10^−6^ (0.0–2899.1), *p* < 0.001), TNFα mRNA level (IQR) (infected 13,657.1 × 10^−6^ (6146.7–27,799.7) vs control 6811.7 (0.0–21,221.2), *p* < 0.001), and IL-6 mRNA level (IQR) (infected 6380.1 × 10^−6^ (2521.6–18,203.2) vs control 1469.7 × 10^−6^ (0.0–12,120.3), *p* < 0.001) were detectable and statistically significantly higher in the infected group when compared with the control group, as shown in Figure 2 and Supplementary Table 3. The findings were reproducible when using the total MPO, ADA, CCL22, TNFα, and IL-6 mRNA levels. Similar findings were observed in multivariate analysis (Supplementary Table 4), with log (MPO/ GAPDH) (odds ratio (OR), 1.423; 95% CI 1.166–1.737, *p* = 0.001), log (ADA/GAPDH) (OR, 5.703; 95% CI 3.424–9.500, *p* < 0.001), log (CCL22/ GAPDH) (OR, 1.735; 95% CI 1.393–2.161, *p* < 0.001), log (TNFα/ GAPDH) (OR, 1.991; 95% CI 1.507–2.629, *p* < 0.001), and log (IL-6/ GAPDH) (OR, 1.583; 95% CI 1.330–1.884, *p* < 0.001) associated with SARS-CoV-2 infection.

**Figure 3.**
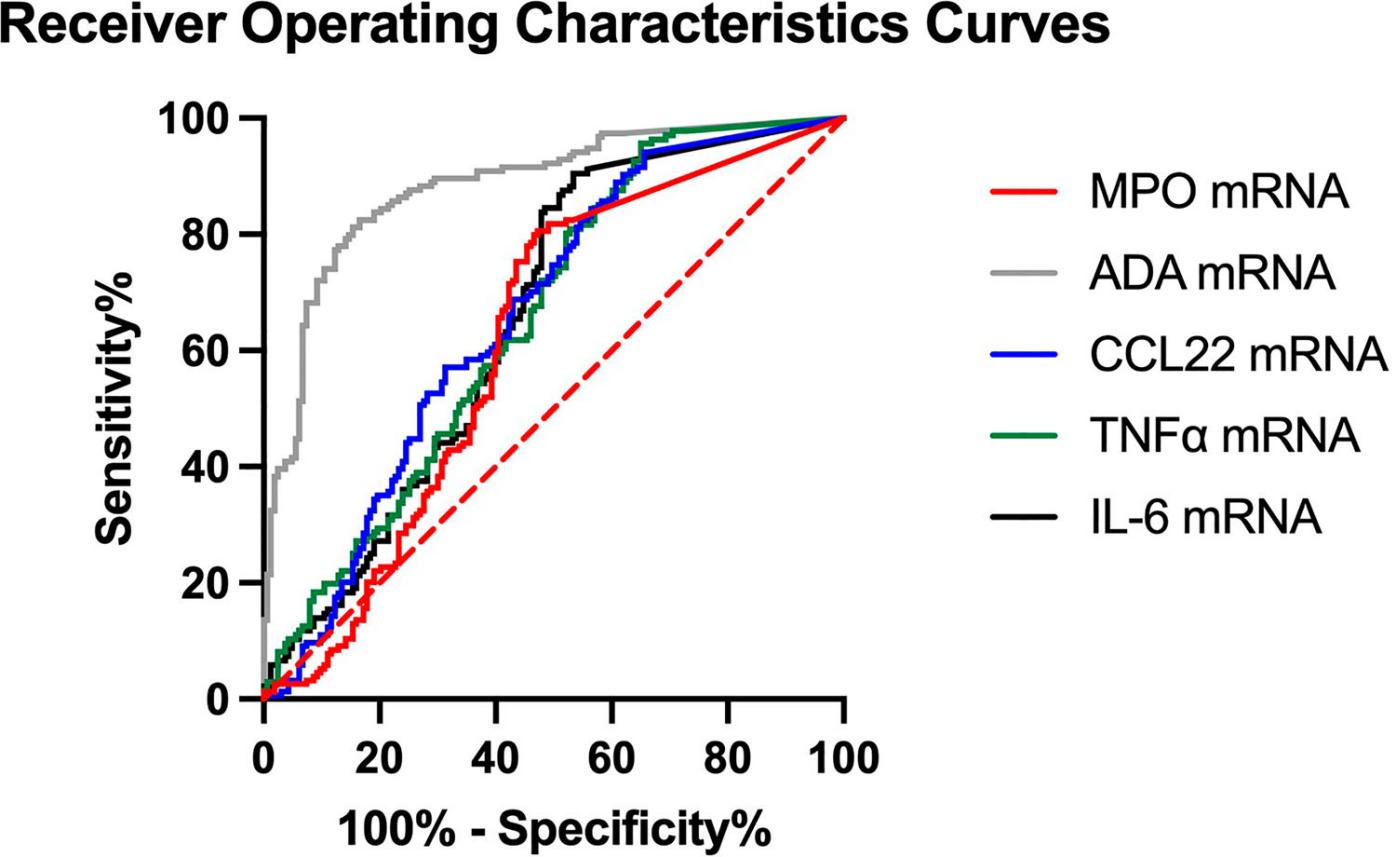
Receiver operating characteristics (ROC) curve of normalized MPO, ADA, CCL22, TNFα, and IL-6 mRNA expression in identification of COVID-19 infected patients. The dotted line represents the line of identity.

ROC curves were plotted for normalized MPO, ADA, CCL22, TNFα, and IL-6 mRNA level, with area under curve being 0.638, 0.897, 0.663, 0.656, and 0.657, respectively, suggesting ADA is better for differentiating infected from uninfected hospitalized patients (Figure 3 and Supplementary Table 5). The cut-off of normalized MPO mRNA level is 7.35 × 10^−5^ with sensitivity 85.3% and specificity 52.8%. The cut-off of normalized ADA mRNA level is 2.37 × 10^−3^ with sensitivity 81.8% and specificity 83.4%. The cut-off of normalized CCL22 mRNA level is 1.01 × 10^−4^ with sensitivity 94.9% and specificity 34.4%. The cut-off of normalized TNFα mRNA level is 2.35 × 10^−3^ with sensitivity 95.6% and specificity 35.0%. The cut-off of normalized IL-6 mRNA level is 9.39 × 10^−4^ with sensitivity 90.4% and specificity 46.6%.

**Figure 4.**
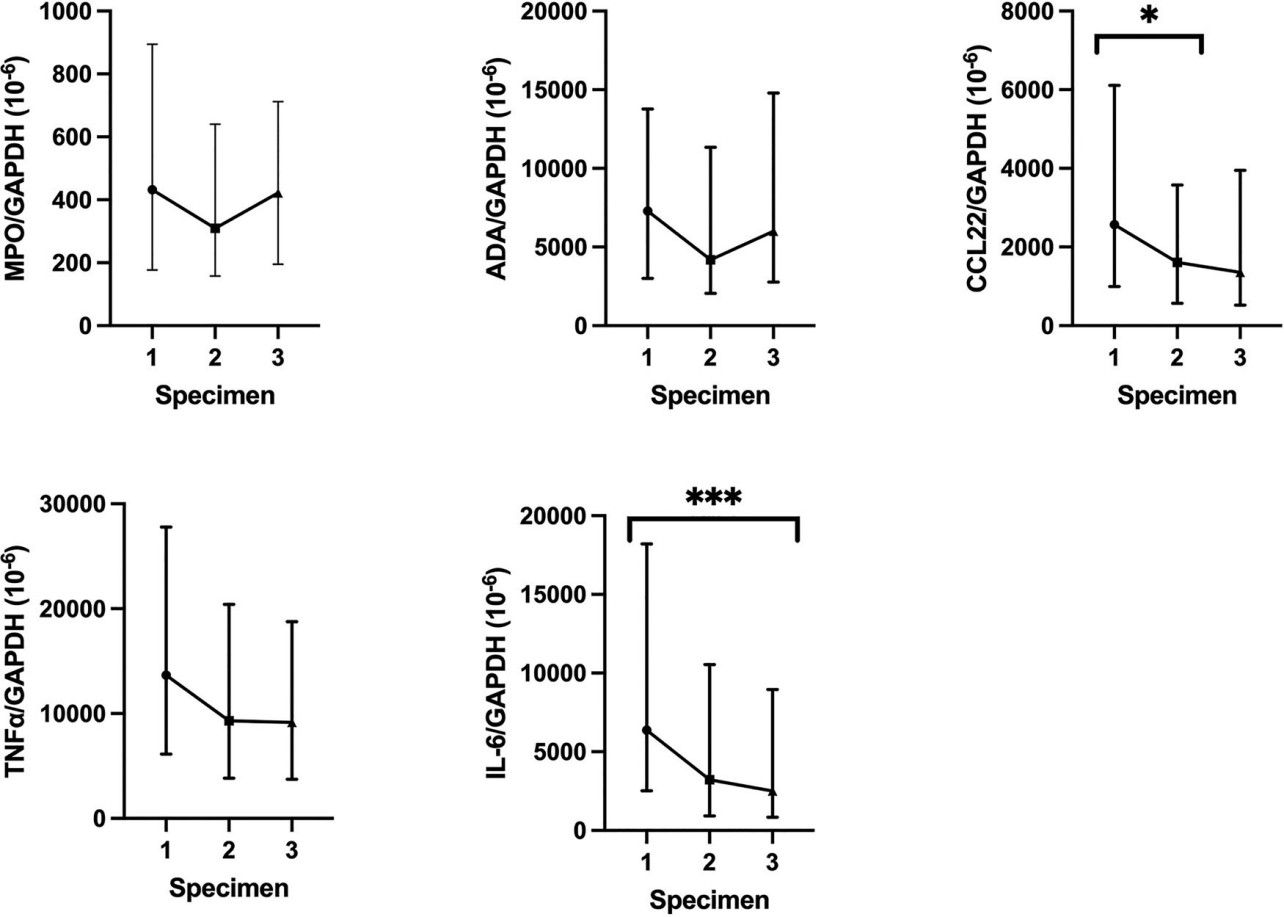
Comparison of normalized MPO, ADA, CCL22, TNFα, and IL-6 mRNA expression in nasopharyngeal swabs between different specimens in COVID-19 patients. The central point represents the median of the samples, the hinges mark the interquartile range. *p < 0.05, **p < 0.005, ***p < 0.001.

For trend of MPO, ADA, CCL22, TNFα, and IL-6 mRNA in serial nasopharyngeal specimens in our COVID-19 patients, MPO, ADA, and TNFα mRNA expression were not associated with any significant trend over 3 time points as shown in Figure 4 and Supplementary Table 6. However, SARS-CoV-2 infection resulted in a significant decrease of CCL22 mRNA level (IQR) during the collection of first and second nasopharyngeal swabs (first 2573.1 × 10^−6^ (994.6–6112.2) vs second 1612.9 × 10^−6^ (568.6–3679.0), *p* = 0.032), as well as a significant decrease of IL-6 mRNA level (IQR) during the collection of first and third nasopharyngeal swabs (first 6380.1 × 10^−6^ (2521.6–18,203.2) vs third 2509.8 × 10^−6^ (843.8–8962.1), *p* < 0.001). Comparison of the MPO, ADA, CCL22, TNFα, and IL-6 mRNA expression between patients with and without completion of COVID-19 vaccines did not reveal any statistically significant differences (Supplementary Table 7).

Of our 154 COVID-19 patients, 95 (61.7%) had severe COVID-19. 46 (48.4%) of the severe COVID-19 patients died, and 13 (13.7%) patients required ICU admission due to COVID-19 (Table 2). Patients with severe COVID-19 were significantly older (83 (IQR 72–90) vs 75 (IQR 64–87), *p* = 0.029) as compared with the 59 (38.3%) mild COVID-19 patients. The Ct values of SARS-CoV-2 PCR were lower among patients with severe disease than those with mild disease, but this difference was not statistically significant (*p* = 0.617). High prevalence of chronic lung disease was observed in the severe COVID-19 group, but the prevalence of other medical comorbidities was similar. Patients with severe COVID-19 had higher incidence of respiratory symptoms when compared with those with mild COVID-19. Comparison of blood parameters on admission revealed higher white blood cell count, neutrophil count, urea and creatinine level, lactate dehydrogenase level, and C-reactive protein in patients with severe COVID-19. More patients with mild COVID-19 had completed the primary series of COVID-19 vaccines. Lymphocyte count (*p* = 0.451) was similar between the two groups.

**Table 2.** Clinical characteristics and outcome of all COVID-19 infected patients classified according to severity of disease. Data are expressed as *n* (%) or median (inter-quartile range), unless otherwise stated. * *p* < 0.05.

	Mild cases	Severe cases	*p*-value
*Total number of cases*	59	95	
*Age – median (Interquartile range)*	75 (64–87)	83 (72–90)	0.029*
*Female*	32 (54.2%)	45 (47.4%)	0.254
*Ct value of first NPS specimen*	26.5 (20.8–32.3)	25.7 (22.0–29.9)	0.617
*Past Medical History*			
* Hypertension*	29 (49.2%)	55 (57.9%)	0.186
* Diabetes Mellitus*	20 (33.9%)	33 (34.7%)	0.529
* Hyperlipidaemia*	16 (27.1%)	27 (28.4%)	0.506
* Ischaemic Heart Disease*	15 (25.5%)	24 (25.3%)	0.564
* Chronic Renal Impairment*	17 (28.8%)	24 (25.3%)	0.381
* Cerebral Vascular Accident*	12 (20.3%)	23 (24.2%)	0.363
* Obesity*	0 (0.0%)	1 (1.1%)	0.617
* Chronic Liver Disease*	6 (10.2%)	7 (7.4%)	0.372
* Chronic Lung Disease*	4 (6.8%)	17 (17.9%)	0.040*
* Solid Organ Malignancy*	9 (15.3%)	9 (9.5%)	0.203
* Haematological Malignancy*	2 (3.4%)	8 (8.4%)	0.187
* Solid Organ Transplantation*	3 (5.1%)	6 (6.3%)	0.525
* Haematological Transplantation*	0 (0.0%)	0 (0.0%)	–
* Autoimmune Disease*	4 (6.8%)	8 (8.4%)	0.485
* Immunosuppressant*	11 (18.6%)	19 (20.0%)	0.505
*Clinical Presentation*			
* Fever*	26 (44.1%)	46 (48.4%)	0.360
* Cough*	20 (33.9%)	48 (50.5%)	0.031*
* Sputum*	9 (15.3%)	31 (32.6%)	0.012*
* Shortness of breath*	15 (25.4%)	68 (71.6%)	<0.001*
* Chest pain*	6 (10.2%)	3 (3.2%)	0.075
* Gastrointestinal Symptoms*	5 (8.5%)	7 (7.4%)	0.515
*Blood Parameters on admission – median (interquartile range)*			
* Haemoglobin, g/dL*	10.6 (8.9–13.1)	11.5 (9.5–13.1)	0.292
* White Blood Cell,* × 10^9^/L	6.0 (3.9–9.4)	8.4 (6.2–12.9)	0.003*
* Neutrophil,* × 10^9^/L	4.3 (3.0–7.6)	7.0 (4.5–10.8)	0.001*
* Lymphocyte,* × 10^9^/L	0.9 (0.5–1.3)	0.8 (0.4–1.2)	0.451
* Platelet,* × 10^9^/L	201 (129–285)	191 (143–260)	0.707
* Sodium, mmol/L*	135 (132–139)	136 (132–142)	0.172
* Potassium, mmol/L*	3.9 (3.5–4.3)	4.1 (3.6–4.6)	0.083
* Urea, mmol/L*	7.6 (4.7–11.3)	10.2 (5.8–23.0)	0.021*
* Creatinine, mmol/L*	91 (69–139)	120 (77–211)	0.014*
* Alkaline Phosphatase, U/L*	75 (66–124)	89 (67–123)	0.319
* Alanine Transaminase, U/L*	23 (15–40)	26 (17–45)	0.371
* Lactate Dehydrogenase, U/L*	258 (205–364)	417 (283–558)	<0.001*
* C-reactive protein, mg/dL*	4.9 (0.6–8.8)	7.4 (2.5–14.2)	0.006*
*Vaccination Status*			0.009*
* Complete*	23 (39.0%)	19 (20.0%)	
* Incomplete/Unvaccinated*	36 (61.0%)	76 (80.0%)	

**Figure 5.**
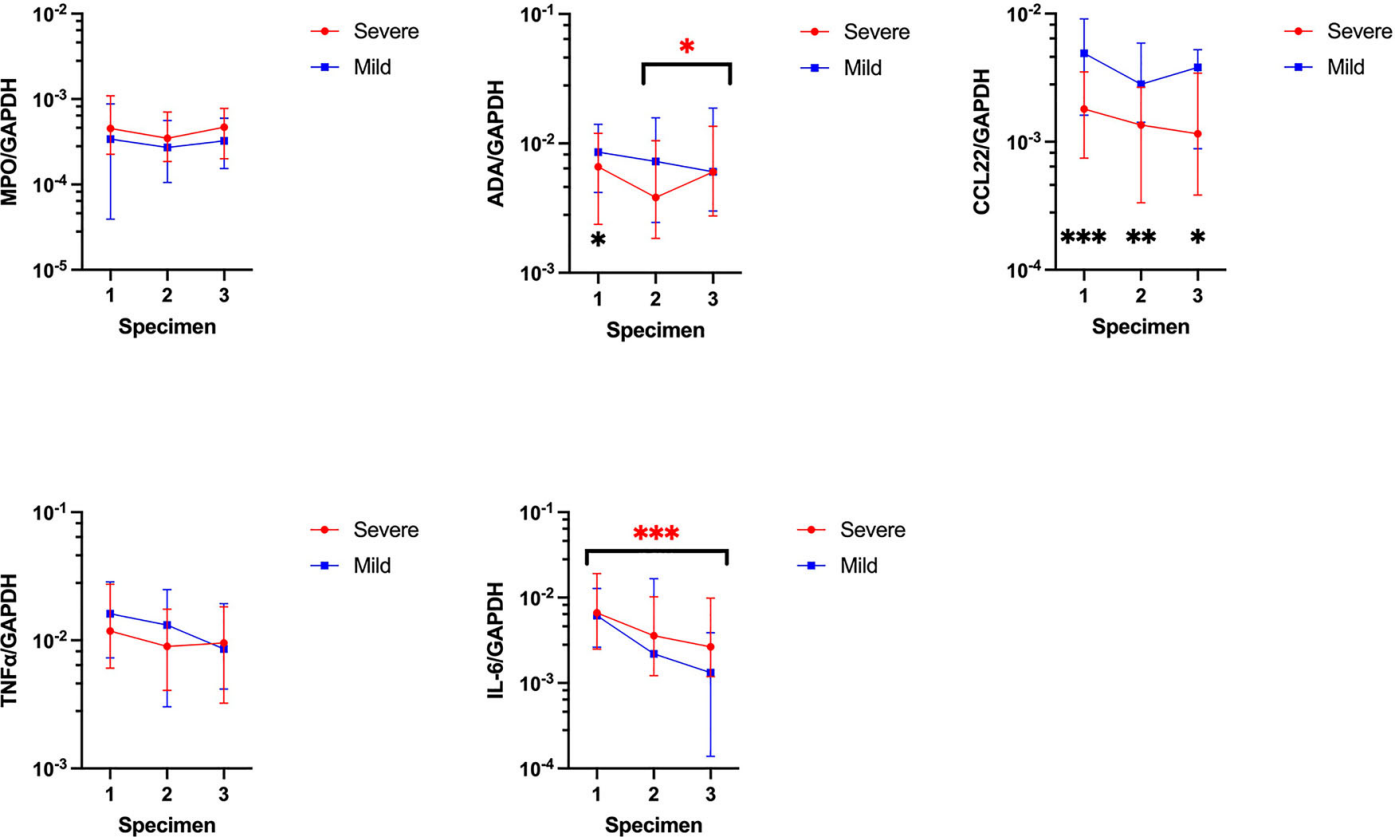
Comparison of normalized MPO, ADA, CCL22, TNFα, and IL-6 mRNA expression in nasopharyngeal swabs between different specimens in patients with mild and severe COVID-19. The central point represents the median of the samples, the hinges mark the interquartile range. *p < 0.05, **p < 0.005, ***p < 0.001.

Expression of MPO and TNFα mRNA in the nasopharyngeal swabs showed no significant differences between patients with mild and severe COVID-19. A similar observation was noted for both ADA and IL-6 mRNA except an increase in expression between the second and third nasopharyngeal specimens in severe COVID-19 patients for ADA (IQR) (second specimen: 3817.5 × 10^−6^ (1846.0–10,472.4) vs third specimen: 6013.0 × 10^−6^ (2748.4–13,545.8), *p* = 0.049), and a decrease in expression between the first and third nasopharyngeal specimens in severe COVID-19 patients for IL-6 (IQR) (first specimen: 6612.9 × 10^−6^ (2491.1–19,090.0) vs third specimen: 2649.5 × 10^−6^ (1181.8–9838.7), *p* < 0.001). For CCL22 mRNA, there were no significant differences in expression among the serial specimens collected from COVID-19 patients; however, CCL22 mRNA expression (IQR) was statistically significantly lower in patients with severe COVID-19 versus those with mild COVID-19 as shown in Figure 5 and Supplementary Table 8 (first specimen: 1779.3 × 10^−6^ (746.9–3514.7) vs 4914.2 × 10^−6^ (1612.3–9114.2), *p* < 0.001; second specimen: 1350.3 × 10^−6^ (334.8–2668.2) vs 2812.3 × 10^−6^ (1421.5–5892.1), *p* = 0.003; third specimen: 1156.1 × 10^−6^ (384.0–3435.2) vs 3814.3 × 10^−6^ (881.4–5240.0), *p* = 0.032).

On multivariate analysis for severity of COVID-19 infection, raised LDH, cough, and shortness of breath were associated with severe COVID-19. History of completion of vaccination and higher log (CCL22/GAPDH) ratio in nasopharyngeal swabs (OR 0.211; 95% CI 0.060–0.746, *p* = 0.016) were associated with mild COVID-19 as shown in Table 3. C-reactive protein was excluded by backward stepwise regression model in the multivariate analysis.

**Table 3.** Multivariate analysis of severe COVID-19 Infection. Mean of CCL22/GAPDH from each patient was obtained, and logarithmic transformation of the ratio was performed for easy interpretation of odds ratio generated by logistic regression. * *p* < 0.05.

Parameters	Multivariate model OR	*p*-value
*Age*	–	–
*Log (Average CCL22/ GAPDH)*	0.211 (0.060–0.746)	0.016*
*Chronic Lung Disease*	7.771 (0.593–101.872)	0.118
*Leucocytosis (WCC > 9.93 × *10^9^/L*)*	–	–
*Neutrophilia (ANC > 7.42 × *10^9^/L*)*	3.220 (0.883–11.736)	0.076
*Urea > 4.4 mmol/L*	–	–
*Creatinine > 83 umol/L*	–	–
*LDH > 221 U/L*	10.146 (2.275–45.252)	0.002*
*CRP > 0.76 mg/dL*	–	–
*Cough*	4.617 (1.230–17.328)	0.023*
*Sputum*	4.465 (0.710–28.094)	0.111
*Shortness of breath*	13.742 (3.938–47.962)	<0.001*
*Completed vaccination*	0.167 (0.035–0.794)	0.024*

## Discussion

We analysed the serial MPO, ADA, CCL22, TNFα, and IL-6 mRNA expression in nasopharyngeal specimens of 154 COVID-19 and 163 control hospitalized patients in the fifth wave of COVID-19 in Hong Kong. We noticed that the mortality rate of our retrospective study cohort (29.9%) was higher than the usual quoted mortality rate of COVID-19 [27]. It is because we could no longer adopt a hospital-based approach by admitting all COVID-19 patients into the healthcare facilities as what we did in the early phase of the COVID-19 pandemic [28]. Although there are significant differences in terms of age, co-morbidities, and blood parameters, multivariate analysis still showed that the expression of MPO, ADA, CCL22, TNFα, and IL-6 mRNA in nasopharyngeal specimens is higher in COVID-19 patients when compared with the control group. This finding signifies the presence of local inflammatory response at the nasopharyngeal mucosa due to SARS-CoV-2 infection, with the interplay of neutrophils, lymphocytes, monocytes, and dendritic cells.

More importantly, ADA mRNA demonstrated the best performance by showing a higher odds ratio in multivariate analysis, when compared with traditional cytokine markers TNFα, and IL-6, and white cell markers MPO and CCL22. Our study reported the cut-off of normalized ADA mRNA level of 2.37 × 10^−3^ has a sensitivity 81.8% and specificity 83.4%, suggesting it to be a reliable marker in nasopharyngeal specimens to differentiate between infected and uninfected patients. Analysis of serial nasopharyngeal specimens of COVID-19 patients failed to show a definitive trend of MPO, ADA, and TNFα mRNA expression, but a decrease in CCL22 mRNA expression was observed between the first and second specimens, which may suggest a decrease in response of dendritic cells during initial infection. SARS-CoV-2 was reported to impair dendritic and T cell responses in patients [29]. Furthermore, a decrease in IL-6 mRNA expression observed signifies the reduction of inflammatory responses and gradual recovery of COVID-19.

To further understand the differences in terms of expression of these markers in nasopharyngeal specimens in COVID-19 patients with different severity, further subgroup analysis was performed. Lymphopenia and inflammatory markers such as C-reactive protein were previously thought to be associated with the severity of COVID-19, but similar findings were not observed in our study [30–34]. On the other hand, lower expression of CCL22 mRNA was observed in nasopharyngeal specimens from severe COVID-19 patients when compared to mild patients. CCL22 controls immunity by promoting regulatory T cell communication with dendritic cells, thus plays an important role in the recruitment and regulation of Th2 cells in the respiratory system [19]. The previous study also reveals acute SARS-CoV-2 infection resulted in reduction in broad immune cells including T-lymphocyte, NK cells, monocyte, and dendritic cells, as well as functional impairment of dendritic cells among severe patients [29]. Therefore, reduced expression of CCL22 mRNA in respiratory specimens may suggest impairment of recruitment of dendritic cells in severe COVID-19 patients [35]. In addition, reduction of CCL22 mRNA expression has been consistently observed in specimens taken on different occasions, with several specimens taken at least 1 month from diagnosis of COVID-19. This concurs with previous findings that impairment of dendritic cells could be persistent up to 7 months from the diagnosis of COVID-19 [36], making CCL22 a promising and durable marker for severe COVID-19. Interestingly, there were no correlations observed between viral load and severity of COVID-19. This could reflect that nasopharyngeal swab can only detect local viral load of SARS-CoV-2 at the nasopharyngeal mucosa, but nasopharyngeal cytokine measurement may reflect the change of cytokine response both locally and systemically.

A previous study has demonstrated the use of one-step real-time RT-qPCR for detection of chemokines mRNA in nasopharyngeal swabs, and the predictive value of C–C motif chemokine Ligand 2/Monocyte chemoattractant protein-1 (CCL2/MCP-1) within 5 days of symptom onset in determining the severity of COVID-19 [11]. Our study has several additional merits. Firstly, our study has a larger sample size with 317 patients while the previous study only included 54 participants. Secondly, there were significant overlaps in the gene expression level in healthy controls and COVID-19 patients in TNFα, CCL2, CCL3, TGFβ, and IL-10 in the aforementioned study, while our study demonstrated that MPO, ADA, CCL22, TNFα, and IL-6 mRNA expression were significantly different between the two groups, with ADA mRNA expression showing the best performance with no overlapping between the interquartile ranges, with reasonable sensitivity and specificity in differentiating infected from uninfected patients.

There are several limitations to our study. Patients with asymptomatic or milder COVID-19 who do not necessitate hospitalization were not included in this study due to StayHomeSafe Scheme implemented during that period [37]. As our study only included adult patients, effect of MPO, ADA, CCL22, TNFα, and IL-6 mRNA expression in paediatric patients was not examined. Moreover, it is a retrospective study conducted in a cluster of hospitals in Hong Kong, therefore variations in the timing of specimen collection were inevitable. However, our study shows that despite such variation, significant difference in CCL22 mRNA expression was still observed between patients with mild and severe COVID-19 in multivariate analysis. With the policy of universal masking in both community and hospital setting in Hong Kong, the number of cases of other respiratory pathogens dropped significantly as shown in previous studies [38–40]. Furthermore, our study focused on the fifth wave with Omicron variant being the predominant variant circulating in Hong Kong. It is uncertain whether similar changes in MPO, ADA, CCL22, TNFα, and IL-6 expression can be observed in other respiratory viruses and other non-Omicron variants of SARS-CoV-2. Further studies are required to see if the expression levels of MPO, ADA, and CCL22 may precede the detection of viral load in fresh nasopharyngeal specimens.

## Conclusion

In summary, the present study demonstrates the increase in nasopharyngeal MPO, ADA, CCL22, TNFα, and IL-6 mRNA expression in COVID-19 patients. More importantly, we discovered that ADA mRNA expression has the best discriminating power when compared with the other markers by multivariate analysis. Furthermore, lower nasopharyngeal CCL22 mRNA expression is associated with severe COVID-19. This finding concurs with previous studies on the impairment of dendritic cells in severe COVID-19. This also serves as an important and durable marker for stratification of severity of cases using the readily available nasopharyngeal specimens.

## Supplementary Material

Supplemental Material

## Data Availability

The data that support the findings of this study are available from the corresponding author, K.Y. Yuen, upon reasonable request.
