# Supplementary material for: Correlations of Myeloperoxidase (MPO), Adenosine deaminase (ADA), C–C motif chemokine 22 (CCL22), Tumour necrosis factor alpha (TNFα) and Interleukin-6 (IL-6) mRNA expression in the nasopharyngeal specimens with the diagnosis and severity of SARS-CoV-2 infections
Source: Emerg Microbes Infect. 2023 Jan 2;12(1):2157338. doi: 10.1080/22221751.2022.2157338 (PMC9809351; doi:10.1080/22221751.2022.2157338)
Supplement: Supplemental Material [file TEMI_A_2157338_SM2725.docx]

Supplementary Table 1. Primer and probe sequences of the real-time RT-PCR assays in this study

| **Target** | **Primer/probe** | **Sequence (5’ to 3’)** |
| --- | --- | --- |
|  |  |  |
| MPO | Forward  Reverse  Probe | CTTCGTCACTGGCGTCAACT  GTTGATCTGGTTGCGGATG  FAM-ATCAAGAACCAAGCCGACTGCAT-IABkFQ |
| ADA | Forward  Reverse | AGAACTGCATGTCCACCTAGA  AGCAGGCATGTAGTAGTCAAACTT |
|  | Probe | FAM-ATCAAGCCTGAAACCATCTTATACTATGGCA-IABkFQ |
|  |  |  |
| CCL22 | Forward  Reverse | GCCGTGATTACGTCCGTTAC  CCACGGTCATCAGAGTAGGC |
|  | Probe | FAM-TAACCTTCAGGGATAAGGAGATCTGT-IABkFQ |
|  |  |  |
| TNFα | Forward | CTCTTCTGCCTGCTGCACTT |
|  | Reverse | TGGGCTACAGGCTTGTCACT |
|  | Probe | FAM-ATGATCTGACTGCCTGGGCC-IABkFQ |
|  |  |  |
| IL-6 | Forward | GACAGCCACTCACCTCTTCA |
|  | Reverse | TTTTCTGCCAGTGCCTCTTT |
|  | Probe | FAM-ATCCTCGACGGCATCTCAGC-IABkFQ |
|  |  |  |
| GAPDH | Forward  Reverse | GAAGGTGAAGGTCGGAGT  GAAGATGGTGATGGGATTTC |
|  | Probe | Cy5-CAAGCTTCCCGTTCTCAGCC-BHQ2 |
|  |  |  |

Supplementary Table 2. Comparison of normalized and total MPO, ADA and CCL22 mRNA expression in nasopharyngeal swabs with different extraction time in uninfected individuals. *p < 0.05, **p < 0.005, ***p < 0.001.

Normalized cytokine markers

| **Total patients**  **(n = 166)** | **MPO/GAPDH x 10^-6^**  **Median (IQR)** | **ADA/GAPDH x 10^-6^**  **Median (IQR)** | **CCL22/GAPDH x 10^-6^**  **Median (IQR)** |
| --- | --- | --- | --- |
| **Extracted within 1 day**  **(n = 104)** | 115.8 (0.0 – 1063.6) | 336.0 (0.0 – 1419.3) | 661.4 (0.0 – 2275.0) |
| **Extracted after 1 day**  **(n = 62)** | 0.0 (0.0 – 0.0) | 0.0 (0.0 – 628.6) | 0.0 (0.0 – 0.0) |
| **p-value** | < 0.001 *** | 0.013 * | 0.001 ** |

Total cytokine markers

| **Total patients**  **(n = 166)** | **MPO (copies/reaction)**  **Median (IQR)** | **ADA (copies/reaction)**  **Median (IQR)** | **CCL22 (copies/reaction)**  **Median (IQR)** |
| --- | --- | --- | --- |
| **Extracted within 1 day**  **(n = 104)** | 8.8 (0.0 – 37.8) | 19.5 (0.0 – 72.3) | 29.8 (0.0 – 113.4) |
| **Extracted after 1 day**  **(n = 62)** | 0.0 (0.0 – 0.0) | 0.0 (0.0 – 29.0) | 0.0 (0.0 – 0.0) |
| **p-value** | < 0.001 *** | 0.016 * | 0.003 ** |

Supplementary Figure 1. Distribution of specimen collection time with respect to the day of first diagnosis of COVID-19. First diagnosis was defined by detection of SARS-CoV-2 RNA by reverse transcription polymerase chain reaction (RT-PCR) or SARS-CoV-2 antigen by rapid antigen test. One specimen was collected 279 days after first diagnosis of COVID-19 was not included in this figure.


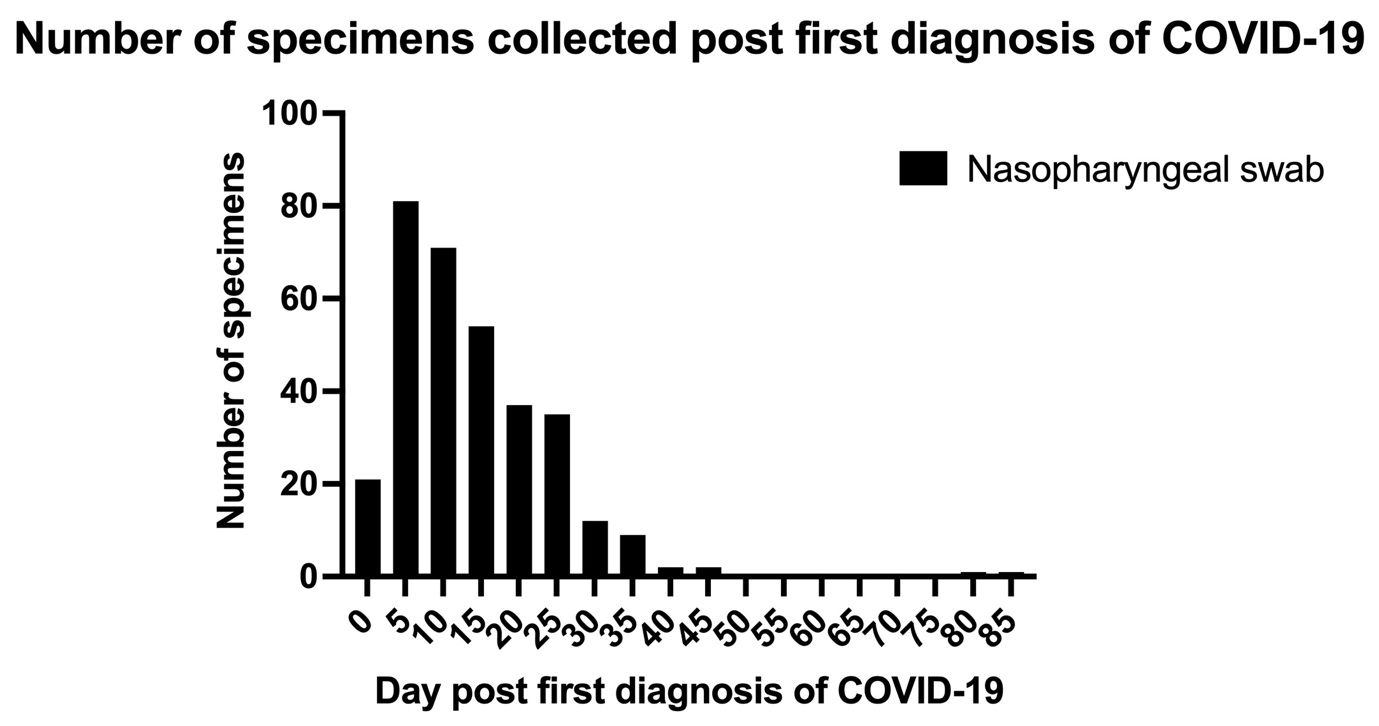


Supplementary Table 3. Comparison of normalized and total MPO, ADA, CCL22, TNFα, and IL-6 mRNA expression in nasopharyngeal swabs between COVID-19 infected and uninfected individuals. *p < 0.05, **p < 0.005, ***p < 0.001.

Normalized cytokine markers

| **Total patients**  **(n = 315)** | **MPO/GAPDH x 10^-6^**  **Median (IQR)** | **ADA/GAPDH x 10^-6^**  **Median (IQR)** | **CCL22/GAPDH x 10^-6^**  **Median (IQR)** | **TNFα/GAPDH x 10^-6^**  **Median (IQR)** | **IL-6/GAPDH x 10^-6^**  **Median (IQR)** |
| --- | --- | --- | --- | --- | --- |
| **Infected patients**  **(n = 154)** | 431.8 (177.3 – 894.3) | 7283.4 (3001.8 – 13768.7) | 2573.1 (994.6 – 6112.2) | 13657.1 (6146.7 – 27799.7) | 6380.1 (2521.6 – 18203.2) |
| **Uninfected patients**  **(n = 163)** | 55.4 (0.0 – 832.3) | 763.3 (0.0 – 1960.2) | 1046.9 (0.0 – 2899.1) | 6811.7 (0.0 – 21221.2) | 1469.7 (0.0 – 12120.3) |
| **p-value** | < 0.001 *** | < 0.001 *** | < 0.001 *** | < 0.001 *** | < 0.001 *** |

Total cytokine markers

| **Total patients**  **(n = 315)** | **MPO (copies/reaction)**  **Median (IQR)** | **ADA (copies/reaction)**  **Median (IQR)** | **CCL22 (copies/reaction)**  **Median (IQR)** | **TNFα**  **(copies/reaction)**  **Median (IQR)** | **IL-6**  **(copies/reaction)**  **Median (IQR)** |
| --- | --- | --- | --- | --- | --- |
| **Infected patients**  **(n = 154)** | 61.6 (17.5 – 333.2) | 855.7 (350.2 – 4618.0) | 462.4 (159.8 – 1728.1) | 2630.4 (813.7 – 10196.4) | 1102.6 (295.3 – 6286.1) |
| **Uninfected patients**  **(n = 163)** | 7.7 (0.0 – 36.6) | 29.6 (0.0 – 148.3) | 41.9 (0.0 – 283.4) | 204.9 (0.0 – 1185.2) | 98.7 (0.0 – 875.9) |
| **p-value** | < 0.001 *** | < 0.001 *** | < 0.001 *** | < 0.001 *** | < 0.001 *** |

Supplementary Table 4. Multivariate analysis of COVID-19 Infection. Logarithmic transformation of all ratios (i.e. MPO/GAPDH, ADA/GAPDH, CCL22/GAPDH, TNFα /GAPDH, and IL-6/GAPDH) were performed for easy interpretation of odds ratio generated by logistic regression. * p < 0.05

| **Parameters** | **Multivariate Model OR** | **p-value** |
| --- | --- | --- |
| ***Chronic Renal Impairment*** | - | - |
| ***Chronic Liver Disease*** | - | - |
| ***Autoimmune disease*** | 6.764 (0.752 – 60.861) | 0.88 |
| ***Immunosuppressant*** | - | - |
| ***Mortality*** | 7.075 (2.900 – 17.260) | < 0.001* |
| ***Anaemia (Hb < 13.3 g/dL for male, < 11.5 g/dL for female)*** | 0.502 (0.275 – 0.914) | 0.024* |
| ***Lymphopenia (< 1.15* x 10^9^/L*)*** | 2.113 (1.212 – 3.685) | 0.008* |
| ***Thrombocytopenia (< 167* x 10^9^/L*)*** | - | - |
| ***Hyponatraemia (< 136 mmol/L)*** | 2.779 (1.604 – 4.817) | < 0.001* |
| ***Hypokalaemia (< 3.6 mmol/L)*** | - | - |
| ***Log (MPO/GAPDH)*** | 1.423 (1.166 – 1.737) | 0.001* |
| ***Completed vaccination*** | 0.314 (0.181 – 0.545) | < 0.001* |

| **Parameters** | **Multivariate Model OR** | **p-value** |
| --- | --- | --- |
| ***Chronic Renal Impairment*** | - | - |
| ***Chronic Liver Disease*** | - | - |
| ***Autoimmune disease*** | - | - |
| ***Immunosuppressant*** | - | - |
| ***Mortality*** | 23.328 (5.787 – 94.035) | < 0.001* |
| ***Anaemia (Hb < 13.3 g/dL for male, < 11.5 g/dL for female)*** | - | - |
| ***Lymphopenia (< 1.15* x 10^9^/L*)*** | 2.524 (1.274 – 4.998) | 0.008* |
| ***Thrombocytopenia (< 167* x 10^9^/L*)*** | 2.153 (0.950 – 4.879) | 0.066 |
| ***Hyponatraemia (< 136 mmol/L)*** | 2.821 (1.410 – 5.643) | 0.003* |
| ***Hypokalaemia (< 3.6 mmol/L)*** | - | - |
| ***Log (ADA/GAPDH)*** | 5.703 (3.424 – 9.500) | < 0.001* |
| ***Completed vaccination*** | 0.270 (0.136 – 0.538) | < 0.001* |

| **Parameters** | **Multivariate Model OR** | **p-value** |
| --- | --- | --- |
| ***Chronic Renal Impairment*** | - | - |
| ***Chronic Liver Disease*** | - | - |
| ***Autoimmune disease*** | 8.620 (0.833 – 89.234) | 0.071 |
| ***Immunosuppressant*** | - | - |
| ***Mortality*** | 7.451 (3.023 – 18.364) | < 0.001* |
| ***Anaemia (Hb < 13.3 g/dL for male, < 11.5 g/dL for female)*** | 0.449 (0.239 – 0.842) | 0.013* |
| ***Lymphopenia (< 1.15* x 10^9^/L*)*** | 2.362 (1.336 – 4.175) | 0.003* |
| ***Thrombocytopenia (< 167* x 10^9^/L*)*** | - | - |
| ***Hyponatraemia (< 136 mmol/L)*** | 2.648 (1.497 – 4.685) | 0.001* |
| ***Hypokalaemia (< 3.6 mmol/L)*** | - | - |
| ***Log (CCL22/GAPDH)*** | 1.735 (1.393 – 2.161) | < 0.001* |
| ***Completed vaccination*** | 0.337 (0.191 – 0.594) | < 0.001* |

| **Parameters** | **Multivariate Model OR** | **p-value** |
| --- | --- | --- |
| ***Chronic Renal Impairment*** | - | - |
| ***Chronic Liver Disease*** | 6.378 (0.992 – 41.011) | 0.051 |
| ***Autoimmune disease*** | - | - |
| ***Immunosuppressant*** | - | - |
| ***Mortality*** | 7.403 (2.890 – 18.958) | < 0.001* |
| ***Anaemia (Hb < 13.3 g/dL for male, < 11.5 g/dL for female)*** | - | - |
| ***Lymphopenia (< 1.15* x 10^9^/L*)*** | 3.018 (1.674 – 5.440) | < 0.001* |
| ***Thrombocytopenia (< 167* x 10^9^/L*)*** | - | - |
| ***Hyponatraemia (< 136 mmol/L)*** | 2.476 (1.370 – 4.476) | 0.030* |
| ***Hypokalaemia (< 3.6 mmol/L)*** | - | - |
| ***Log (TNFα /GAPDH)*** | 1.991 (1.507 – 2.629) | < 0.001* |
| ***Completed vaccination*** | 0.337 (0.186 – 0.611) | < 0.001* |

| **Parameters** | **Multivariate Model OR** | **p-value** |
| --- | --- | --- |
| ***Chronic Renal Impairment*** | - | - |
| ***Chronic Liver Disease*** | 5.772 (1.140 – 29.228) | 0.034* |
| ***Autoimmune disease*** | - | - |
| ***Immunosuppressant*** | - | - |
| ***Mortality*** | 7.885 (3.033 – 20.494) | < 0.001* |
| ***Anaemia (Hb < 13.3 g/dL for male, < 11.5 g/dL for female)*** | 0.522 (0.271 – 1.004) | 0.051 |
| ***Lymphopenia (< 1.15* x 10^9^/L*)*** | 2.950 (1.624 – 5.360) | 0.001* |
| ***Thrombocytopenia (< 167* x 10^9^/L*)*** | - | - |
| ***Hyponatraemia (< 136 mmol/L)*** | 2.747 (1.523 – 4.956) | 0.001* |
| ***Hypokalaemia (< 3.6 mmol/L)*** | - | - |
| ***Log (IL-6/GAPDH)*** | 1.583 (1.330 – 1.884) | < 0.001* |
| ***Completed vaccination*** | 0.380 (0.211 – 0.685) | 0.001* |

Supplementary Table 5. Details of receiver operating characteristics (ROC) curve of normalized MPO, ADA, CCL22, TNFα, and IL-6 mRNA expression in nasopharyngeal swabs in infected and uninfected patients

|  | **Area under curve (AUC)** | **Optimal cut-off (normalized)** | **Sensitivity** | **Specificity** |
| --- | --- | --- | --- | --- |
| **MPO** | 0.638 | 7.35 x 10^-5^ | 85.3% | 52.8% |
| **ADA** | 0.897 | 2.37 x 10^-3^ | 81.8% | 83.4% |
| **CCL22** | 0.663 | 1.01 x 10^-4^ | 94.9% | 34.4% |
| **TNFα** | 0.656 | 2.35 x 10^-3^ | 95.6% | 35.0% |
| **IL-6** | 0.657 | 9.39 x 10^-4^ | 90.4% | 46.6% |

Supplementary Table 6. Comparison of MPO, ADA, CCL22, TNFα, and IL-6 mRNA expression in nasopharyngeal swabs between different specimens in COVID-19 patients. *p < 0.05, **p < 0.005, ***p < 0.001.

|  | **MPO/GAPDH x 10^-6^**  **Median (IQR)** | **ADA/GAPDH x 10^-6^**  **Median (IQR)** | **CCL22/GAPDH x 10^-6^**  **Median (IQR)** | **TNFα/GAPDH x 10^-6^**  **Median (IQR)** | **IL-6/GAPDH x 10^-6^**  **Median (IQR)** |
| --- | --- | --- | --- | --- | --- |
| **1^st^ specimen** | 431.8 (177.3 – 894.3) | 7283.4 (3001.8 – 13768.7) | 2573.1 (994.6 – 6112.2) | 13657.1 (6146.7 – 27799.7) | 6380.1 (2521.6 – 18203.2) |
| **p-value on difference between the 1^st^ and 2^nd^ specimen** | 0.402 | 0.759 | 0.032 * | 0.899 | 0.109 |
| **2^nd^ specimen** | 309.8 (157.6 – 640.1) | 4190.4 (2065.9 – 11339.2) | 1612.9 (568.6 – 3679.0) | 9302.1 (3826.0 – 20403.8) | 3217.9 (926.7 – 10540.3) |
| **p-value on difference between the 2^nd^ and 3^rd^ specimen** | 0.236 | 0.387 | 0.969 | 0.653 | 0.186 |
| **3^rd^ specimen** | 421.9 (195.0 – 711.9) | 6013.0 (2777.9 – 14794.3) | 1358.9 (524.1 – 3946.7) | 9150.8 (3741.8 – 18753.5) | 2509.8 (843.8 – 8962.1) |
| **p-value on difference between the 1^st^ and 3^rd^ specimen** | 0.965 | 0.871 | 0.102 | 0.092 | <0.001*** |

Supplementary Table 7. Comparison of MPO, ADA, CCL22, TNFα, and IL-6 mRNA expression in nasopharyngeal swabs in COVID-19 patients . *p < 0.05, **p < 0.005, ***p < 0.001.

| **Infected**  **(n = 154)** | **MPO/GAPDH x 10^-6^**  **Median (IQR)** | **ADA/GAPDH x 10^-6^**  **Median (IQR)** | **CCL22/GAPDH x 10^-6^**  **Median (IQR)** | **TNFα/GAPDH x 10^-6^**  **Median (IQR)** | **IL-6/GAPDH x 10^-6^**  **Median (IQR)** |
| --- | --- | --- | --- | --- | --- |
| **Incomplete vaccination**  **(n = 112)** | 379.9 (154.6 – 868.3) | 6934.0 (2585.4 – 12319.0) | 22488.5 (947.7 – 5642.6) | 11815.2 (5996.2 – 24158.2) | 5914.7 (2461.0 – 18887.9) |
| **Complete vaccination**  **(n = 42)** | 463.6 (236.3 – 1100.0) | 9746.7 (5441.8 – 16422.9) | 2802.5 (1099.6 – 7724.7) | 20257.9 (8880.0 – 40230.1) | 7279.9 (2763.0 – 14844.5) |
| **p-value** | 0.528 | 0.058 | 0.641 | 0.055 | 0.992 |

Supplementary Table 8. Comparison of MPO, ADA, CCL22, TNFα, and IL-6 mRNA expression in nasopharyngeal swabs between different specimens in patients with mild and severe COVID-19. *p < 0.05, **p < 0.005, ***p < 0.001.

| **MPO/GAPDH**  **Median (IQR) x 10^-6^** | **1^st^ specimen** | **2^nd^ specimen** | **3^rd^ specimen** |
| --- | --- | --- | --- |
| **Severe Cases** | 452.0 (225.6 – 1094.6) | 347.7 (185.6 – 704.1) | 470.1 (200.3 – 776.6) |
| **Mild Cases** | 340.0 (39.2 – 876.8) | 271.4 (105.4 – 561.2) | 324.2 (153.2 – 598.1) |
| **p-value between specimens in severe cases** | 0.475 (between 1^st^ and 2^nd^ specimen) | 0.337 (between 2^nd^ and 3^rd^ specimen) | 0.636 (between 1^st^ and 3^rd^ specimen) |
| **p-value between specimens in mild cases** | 0.683 (between 1^st^ and 2^nd^ specimen) | 0.472 (between 2^nd^ and 3^rd^ specimen) | 0.435 (between 1^st^ and 3^rd^ specimen) |
| **p-value between mild and severe cases** | 0.181 | 0.264 | 0.270 |

| **ADA/GAPDH**  **Median (IQR) x 10^-6^** | **1^st^ specimen** | **2^nd^ specimen** | **3^rd^ specimen** |
| --- | --- | --- | --- |
| **Severe Cases** | 6592.8 (2367.6 – 11976.4) | 3817.5 (1846.0 – 10472.4) | 6013.0 (2748.4 – 13545.8) |
| **Mild Cases** | 8566.2 (4174.8 – 14027.1) | 7261.7 (2453.6 – 15751.2) | 6038.5 (3003.7 – 18736.0) |
| **p-value between specimens in severe cases** | 0.701 (between 1^st^ and 2^nd^ specimen) | 0.049 * (between 2^nd^ and 3^rd^ specimen) | 0.426 (between 1^st^ and 3^rd^ specimen) |
| **p-value between specimens in mild cases** | 1.000 (between 1^st^ and 2^nd^ specimen) | 0.078 (between 2^nd^ and 3^rd^ specimen) | 0.170 (between 1^st^ and 3^rd^ specimen) |
| **p-value between mild and severe cases** | 0.019* | 0.143 | 0.954 |

| **CCL22/GAPDH**  **Median (IQR) x 10^-6^** | **1^st^ specimen** | **2^nd^ specimen** | **3^rd^ specimen** |
| --- | --- | --- | --- |
| **Severe Cases** | 1799.3 (746.9 – 3514.7) | 1350.3 (334.8 – 2668.2) | 1156.1 (384.0 – 3435.2) |
| **Mild Cases** | 4914.2 (1612.3 – 9114.2) | 2812.3 (1421.5 – 5892.1) | 3814.3 (881.4 – 5240.0) |
| **p-value between specimens in severe cases** | 0.072 (between 1^st^ and 2^nd^ specimen) | 0.689 (between 2^nd^ and 3^rd^ specimen) | 0.220 (between 1^st^ and 3^rd^ specimen) |
| **p-value between specimens in mild cases** | 0.246 (between 1^st^ and 2^nd^ specimen) | 0.528 (between 2^nd^ and 3^rd^ specimen) | 0.215 (between 1^st^ and 3^rd^ specimen) |
| **p-value between mild and severe cases** | < 0.001*** | 0.003 ** | 0.032 * |

| **TNFα/GAPDH**  **Median (IQR) x 10^-6^** | **1^st^ specimen** | **2^nd^ specimen** | **3^rd^ specimen** |
| --- | --- | --- | --- |
| **Severe Cases** | 11815.2 (6052.8 – 27431.3) | 8951.6 (4059.4 – 17484.5) | 9543.8 (3233.1 – 18200.6) |
| **Mild Cases** | 16118.9 (7280.3 – 28580.5) | 13156.4 (3023.5 – 24892.9) | 8554.4 (4162.4 – 19294.2) |
| **p-value between specimens in severe cases** | 0.906 (between 1^st^ and 2^nd^ specimen) | 0.952 (between 2^nd^ and 3^rd^ specimen) | 0.171 (between 1^st^ and 3^rd^ specimen) |
| **p-value between specimens in mild cases** | 0.879 (between 1^st^ and 2^nd^ specimen) | 0.331 (between 2^nd^ and 3^rd^ specimen) | 0.248 (between 1^st^ and 3^rd^ specimen) |
| **p-value between mild and severe cases** | 0.278 | 0.397 | 0.816 |

| **IL-6/GAPDH**  **Median (IQR) x 10^-6^** | **1^st^ specimen** | **2^nd^ specimen** | **3^rd^ specimen** |
| --- | --- | --- | --- |
| **Severe Cases** | 6612.9 (2491.1 – 19090.0) | 3570.1 (1219.1 – 10188.0) | 2649.5 (1181.8 – 9838.7) |
| **Mild Cases** | 6131.2 (2623.3 – 12800.1) | 2189.7 (0.0 – 16660.9) | 1325.0 (138.2 – 3880.2) |
| **p-value between specimens in severe cases** | 0.053 (between 1^st^ and 2^nd^ specimen) | 0.708 (between 2^nd^ and 3^rd^ specimen) | < 0.001*** (between 1^st^ and 3^rd^ specimen) |
| **p-value between specimens in mild cases** | 0.877 (between 1^st^ and 2^nd^ specimen) | 0.064 (between 2^nd^ and 3^rd^ specimen) | 0.062 (between 1^st^ and 3^rd^ specimen) |
| **p-value between mild and severe cases** | 0.417 | 0.345 | 0.101 |
